# Seasonal Differences in Leaf Photoprotective Potential between Adults and Juveniles of Two Mediterranean Perennials with Distinct Growth Forms: A Comparative Field Study

**DOI:** 10.3390/plants12173110

**Published:** 2023-08-30

**Authors:** Christos Chondrogiannis, Kassiani Kotsi, George Grammatikopoulos, Yiola Petropoulou

**Affiliations:** Laboratory of Plant Physiology, Department of Biology, University of Patras, 26504 Patras, Greece; chondroc@tcd.ie (C.C.); grammati@upatras.gr (G.G.)

**Keywords:** Mediterranean plants, photoprotective potential, non-photochemical quenching, electron transport rate, xanthophyll cycle, reproductive maturation

## Abstract

The photosynthetic differences between adult and juvenile Mediterranean plants were previously studied under field conditions, yet the corresponding differentiation of their photoprotective efficiency has not been sufficiently investigated. The present study aims to examine possible differences in the photoprotective potential between adults and juveniles of two native Mediterranean plants with distinct growth forms. Thus, the seasonal variations in individual carotenoids, electron transport rate (ETR), and non-photochemical quenching (NPQ) were monitored in fully exposed mature leaves from adults and juveniles of the winter deciduous tree *Cercis siliquastrum* L. and the evergreen sclerophyllous shrub *Nerium oleander* L. All plants were grown under apparently similar field conditions. In both species, juveniles displayed substantially lower ETR and increased NPQ values than adults in spring, with the differences intensifying during summer drought and diminishing in autumn. Concomitantly, juveniles showed significantly higher chlorophyll-based total carotenoids in spring and summer mainly due to the higher investment in xanthophyll cycle components (VAZ), in combination with an increased mid-day de-epoxidation state (DEPS) and partial retention of zeaxanthin in the dark. In *N. oleander*, although ETR was lower in juveniles during winter, NPQ was extremely low in both ages. In conclusion, juveniles exhibit enhanced photoprotection potential, especially in the summer, due to their reduced photochemical capacity. The photosynthetic superiority of adults during the favorable spring period may be attributed to the needs of the co-existing reproductive effort.

## 1. Introduction

Mediterranean plants face the threat of photosynthesis inhibition as they usually photosynthesize under high light intensities [1]. Photodamage can be severe either during the summer drought period or the coldest periods of winter [2,3]. Short-term impairments in photosynthesis may also occur even during the mild periods of spring and autumn; however, they are moderate and uncertain depending on the fluctuations in local environmental conditions [4,5]. The capture of light and the production of chemical energy via the light-dependent reactions of photosynthesis must be properly coordinated with the corresponding light-independent ones, in which chemical energy is used for sugar production [6]. If any intrinsic or extrinsic factors disturb this balance, thus generating an excess of unutilized absorbed energy, a cascade of intracellular events modifies the structure and function of the photosynthetic machinery [6,7].

Plants growing in Mediterranean-type ecosystems possess a rich repertoire of photoprotective mechanisms against the hazards induced by photoinhibition. Photoprotection includes (a) light absorption-avoiding mechanisms, such as steep leaf angles, leaf rolling, the accumulation of highly reflecting or absorbing pigments in leaf epidermis and/or mesophyll, the presence of highly reflective trichomes, scales and cuticular waxes, and chloroplast movements [8,9,10,11]; and/or (b) the effective biochemical/biophysical consumption of excess light energy, such as smaller antenna sizes, heat dissipation, alternative electron flow paths, and state transitions of photosystems [7,12,13]. Finally, if the production of harmful reactive oxygen species (ROS) cannot be avoided, a more or less effective ROS scavenging system is activated as the last defense mechanism against the oxidation of cellular molecules [14]. The heat dissipation mechanism is the major contributor to the non-photochemical quenching of absorbed energy (NPQ) and is of great importance as it can rapidly adjust the amount of energy conversion into heat under highly variable light conditions during the day, even within hours or minutes [6,15]. The heat dissipation process has been related to xanthophyll cycle activity [16,17]. Under excess light, violaxanthin (V) is de-epoxidized rapidly to zeaxanthin (Z) via the intermediate antheraxanthin (A). Zeaxanthin is the critical molecule for the deactivation of an excited singlet chlorophyll (^1^Chl*) state [18].

Apart from the environmental parameters, photosynthesis is also influenced by developmental leaf changes, which more or less reflect the maturation of the chloroplast and its corresponding structural and functional modifications [19,20]. Additionally, the photosynthetic characteristics of mature leaves can be affected by the increasing age and size of the plant [5,21,22,23,24]. Regarding woody perennials, pre-reproductive individuals are considered juveniles, while fully reproductive ones are considered mature [25]. Chlorophyll concentration, stomatal conductance, mesophyll conductance, electron transport rate, and Rubisco activity are often modified after maturation [5,26,27,28]. However, the magnitude of differences depends on both the plant’s growth form and prevailing environmental conditions, which exhibit strong variations among seasons in regions with Mediterranean climates [5,28,29]. Consequently, the efficiency of photoprotection would also be expected to vary between adults and juveniles. The photoprotective mechanisms in the adults of many Mediterranean species have been extensively studied [27,30,31,32], yet relevant information for their juveniles is scarce [33,34]. Recently, photosynthetic differences between adults and juveniles of *Nerium oleander* and *Cercis siliquastrum* were studied [5,20]. In general, the juveniles of the given species showed lower photosynthetic activity compared to adults during all seasons. The differences were attributed to both the maturation status of the plants and the prevailing environmental conditions. In most previous studies, which were performed on tree species, the light environment of adults and juveniles was quite different because the young seedlings and, subsequently, the juveniles were commonly grown in the forest’s understory and received significantly lower irradiation intensities compared to adults [21,25,35]. On the other hand, the shrub *Nerium oleander* and the small tree *Cercis siliquastrum* usually grow in open habitats where juveniles are not shaded by mature plants. Therefore, any possible differences in photoprotection capacity should not be attributed to acclimation at low or high light intensity [5,20].

The aim of the present work is (a) to reveal possible differences in non-photochemical quenching, xanthophyll cycle, and electron flow activity between the adults and juveniles of two characteristic Mediterranean perennials with distinct growth forms and (b) to attribute these differences to the reproductive maturation of plants and/or to the strong seasonality of the Mediterranean climate. To this purpose, chlorophyll fluorescence measurements in the light-adapted state and chromatographic analyses of the photosynthetic pigments were carried out seasonally on mature leaves from adult and juvenile individuals of the evergreen sclerophyll shrub *N. oleander* and the winter deciduous tree *C. siliquastrum* with pre-leafing flowering.

## 2. Materials and Methods

### 2.1. Plant Material, Experimental Site, and Sampling

Adult (>20 years) and juvenile (<6 years) individuals of two woody species (*Nerium oleander* L. and *Cercis siliquastrum* L.), belonging to different growth forms, were used throughout the study. *N. oleander* (Apocynaceae) is a sclerophyllous evergreen shrub of the Mediterranean flora, possessing leaves with a typical sclerophyll anatomy and a life span extending to more than two years. *C. siliquastrum* (Fabaceae) is a winter deciduous tree, native of the Mediterranean basin (to regions between Western Asia and South-Eastern Europe), producing bright pink flowers in early spring before leaf emergence. New leaves are produced shortly after flowering commencement, having a life span of about 7 months.

**Figure 1:**
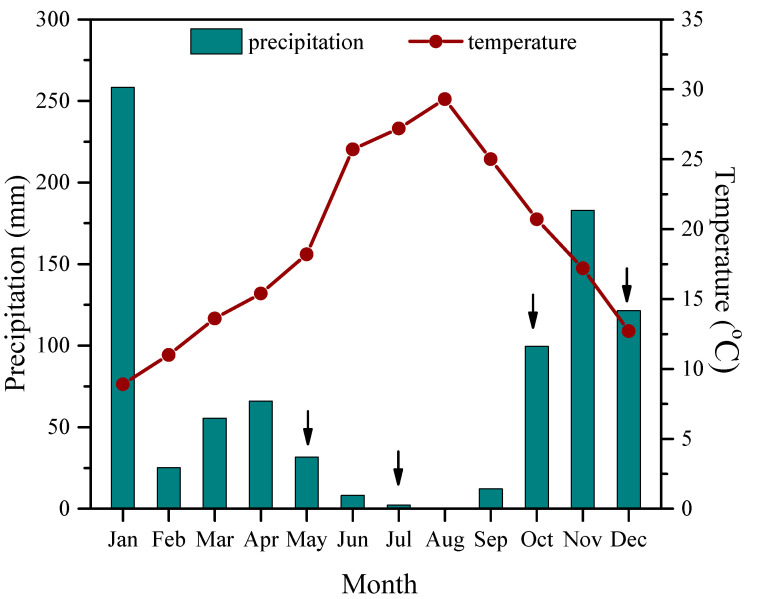
Mean monthly air temperature (°C, red line) and monthly total precipitation (mm, columns) for 2019 in the study area at the Patras University Campus. Arrows denote the measuring dates.

For each species, three representative adult and three juvenile plants were selected from a population for which the year of germination and/or transplantation was known from previous studies of our laboratory, tagged, and used for further experimentation. The selected individuals, in which plant size difference between adults and juveniles was of the minimum possible, were grown in the wild under similar field conditions, in undisturbed areas of the Patras University Campus (38.29° N, 21.79° E, 63 m a.s.l.), and receiving only natural precipitation. Measurements were conducted seasonally, during spring (late-May), summer (mid-July), autumn (late-October), and winter (December) of 2019, on days representative of each distinct season. Environmental conditions at the experimental region were representative of the Mediterranean climate, with a hot dry summer and mildly cold winter (Figure 1). The mean daily temperature was 18.2 °C in spring, 27.2 °C in summer, 20.7 °C in autumn, and 12.7 °C in winter, while in the same months total precipitation was 31.6, 2.2, 99.6, and 121.4 mm, respectively.

Sampling was always performed on cloudless days. Only mature, totally exposed to solar radiation (i.e., south-facing) leaves of the current developmental period were used. In each measuring period, chlorophyll fluorescence measurements were conducted on 2 leaves per individual (i.e., a total of 12 leaves from each species, 6 leaves per age class) from 9 am to 12 am. The following day, leaf harvesting (2 leaves per individual) for photosynthetic pigment determination was performed twice, at pre-dawn and at mid-day, to check possible diurnal VAZ cycle interconversions. The leaves were immediately immersed in liquid nitrogen and transferred to the laboratory, where they were stored at −80 °C until extraction.

### 2.2. Chlorophyll Fluorescence Measurements in the Light Acclimated State

Chlorophyll fluorescence response curves [36] of light adapted, attached to the plant leaves, were recorded with a portable pulse-amplitude modulated fluorometer (MINI-PAM; Walz Effeltrich, Germany), connected to a leaf clip (Leaf-Clip Holder 2030-B; Walz Effeltrich, Germany). The instrument is equipped with a LED light source providing a weak measuring beam (<0.05 μmol m^−2^ s^−1^) plus a white light halogen source producing the actinic light and the saturation pulses (8000 μmol m^−2^ s^−1^, 0.8 s). Leaves were initially darkened with dark clips for 30 min for F_0_ and F_M_ estimation (F_0_ and F_M_ measurement protocol). Then, light intensity increased progressively, from 0 to 2000 μmol m^−2^ s^−1^ PAR and each light curve was composed of eight steps (with a 30 s duration each) using the Leaf-Clip Holder. During the RLC protocol, leaves were shielded from direct sunlight with the help of a black cloth on the corresponding branch of the mature plants or on the top of the juveniles. Consequently, measured leaves were receiving only the instrument internal actinic light of predetermined intensities. The fiberoptics were positioned at 60 degrees to the leaf surface. At each step, the fluorescence before the saturation pulse (F_s_) and the maximum fluorescence during the saturation pulse (F′_M_) were obtained to calculate the photosystem II photochemical efficiency as Φ_PSII_ = (F′_M_ − F_s_/F′_M_), according to Genty et al., 1989 [37]. The apparent linear electron transport rate through PSII was calculated as ETR = Φ_PSII_ × PAR × Abs × 0.5, where PAR is the incident photosynthetically active radiation, Abs is the leaf absorptance (0.85), and 0.5 is a correction factor assuming equal distribution of absorbed photons between the two photosystems. Non-photochemical quenching (NPQ) was calculated as NPQ = (F_M_ − F′_M_)/F′_M_ [38], using the F_M_ value of the initial measurement before the onset of the RLC protocol.

### 2.3. Rapid Light Curve Fitting

In order to compare RLCs between adults and juveniles quantitatively, the ETR_max_, E_k_, and E_m_ parameters were determined. To this aim, an empirical double exponential decay equation with a Marquardt–Levenberg regression algorithm [39] was used to describe the photosynthetic response as a function of light. ETR_max_ (maximum electron transport rate), is related to the maximum photosynthetic capacity. The value of the initial slope (*a*) of the light curve was estimated by calculating the slope of the first three to four data points (initial linear part of the curve). E_k_ is the minimum saturating irradiance and is determined as the interception of the initial linear part of the curve with the maximum electron transport rate. E_m_ is the irradiance at maximum electron transport rate. Data exported from Mini-Pam were further analyzed with v27 IBM SPSS and the model was fitted using MS Excel Solver.

### 2.4. Photosynthetic Pigments

Discs of known diameter were punched out from the middle part of each leaf and the discs of the same individual were frozen in a mortar via adding a small volume of liquid nitrogen to facilitate extraction and prevent any VAZ cycle interconversion. Chlorophylls and carotenoids were extracted in dim light with pure methanol plus a small amount of CaCO_3_ to avoid chlorophyll pheophytinization. After centrifugation at 6000 rpm for 10 min, the supernatant was further cleared by passing through a 0.45 μm syringe filter. Total chlorophylls and carotenoids were measured spectrophotometrically, using a Shimadzu (UV-160A) double-beam spectrophotometer and the concentrations were estimated according to the equations of Wellburn, 1994 [40].

Separation of individual carotenoids was performed with a Shimadzu LC-10 AD HPL chromatograph, equipped with a SIL-HTC autosampler, a CTO-10ACVP column oven, and a non-endcapped Zorbax ODS (4.6 mm 9 25.0 mm) column (Rockland Technologies, Chadds Ford, PA, USA), as previously described [41]. The chromatographic system was calibrated against purified b-carotene, lutein, and zeaxanthin (Extrasynthese, Lyon Nord, France), and a system controller (SCL-10AVP) was used for the control of all components.

Elution was performed isocratically at 1 mL min^−1^ (20 min with acetonitrile:methanol, 85:15 *v*/*v*, and 20 min with methanol:ethyl acetate, 68:32 *v*/*v*), according to Thayer and Björkman [42] as modified by Kyzeridou et al., 2015 [41]. Pigments were detected via measuring absorbance at 445 nm, using a Shimadzu SPDM10AVP UV/Vis photo-diode array detector, and further analyzed by a Shimadzu Class-VP (version 6.1) software package. Each extracted sample was double analyzed in two separate chromatographic runs.

### 2.5. Statistical Analysis

Significance of differences for the measured parameters between adults and juveniles of each species were assessed by independent *t*-test using the SPSS v.27 statistical package (IBM-SPSS Statistics, Armonk, NY, USA). Statistically significant differences (at *p* < 0.05) are indicated by asterisks (between adults and juveniles within each season) or different letters (for adults or juveniles between seasons). The number of independent measurements in each case is given in the legends of tables and figures.

## 3. Results

The photosynthetic and photoprotective potential of mature leaves from adults and juveniles of the two species tested was compared on a seasonal basis. Prevailing environmental conditions at the experimental site were representative of the Mediterranean climate, with a hot dry summer and low temperatures during winter (Figure 1). The highest mean daily temperatures throughout the sampling period were observed in July and August (27.2 and 29.3 °C, respectively), and the lowest in December (12.7 °C). Leaf temperature during the measurement periods was 0.5–1.5 °C higher than the prevailing air temperatures in both adults and juveniles at all seasons. Precipitation in summer was minimal, as the sum of the summer months was only 10.4 mm, while during a ~2 month period preceding spring, autumn, and winter measurements it was adequate, reaching 98, 112, and 304 mm, respectively. Thus, weather conditions in spring and autumn were similar and advantageous for photosynthesis, while in the two adverse seasons plants faced high light intensities combined with summer drought and low winter temperatures, respectively.

### 3.1. Chlorophyll Fluorescence Measurements

**Figure 2:**
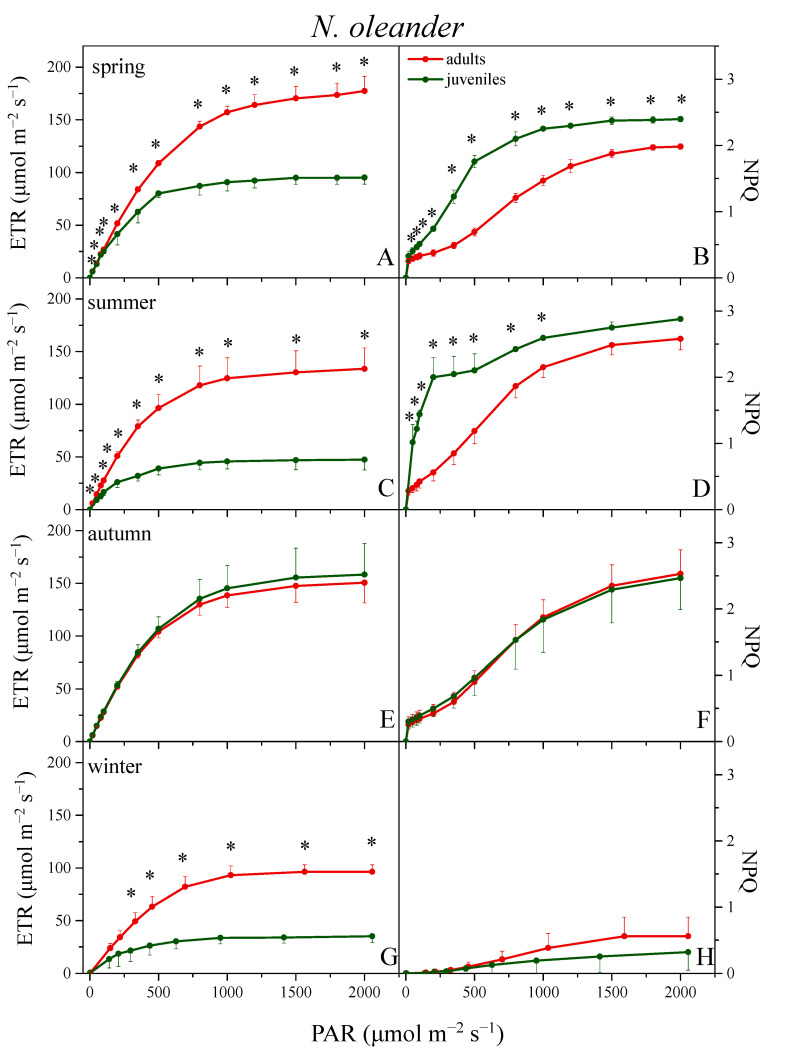
Light response curves of linear electron transport rate (ETR, left panels) and non-photochemical quenching (NPQ, right panels) from adults (red circles) and juveniles (green circles) of *N. oleander* during spring (**A**,**B**), summer (**C**,**D**), autumn (**E**,**F**), and winter (**G**,**H**). Data are means ± SD of 6 measurements (2 measurements/individual). Asterisks denote statistically significant differences (*p* < 0.05) between adults and juveniles at each light intensity within each season.

**Figure 3:**
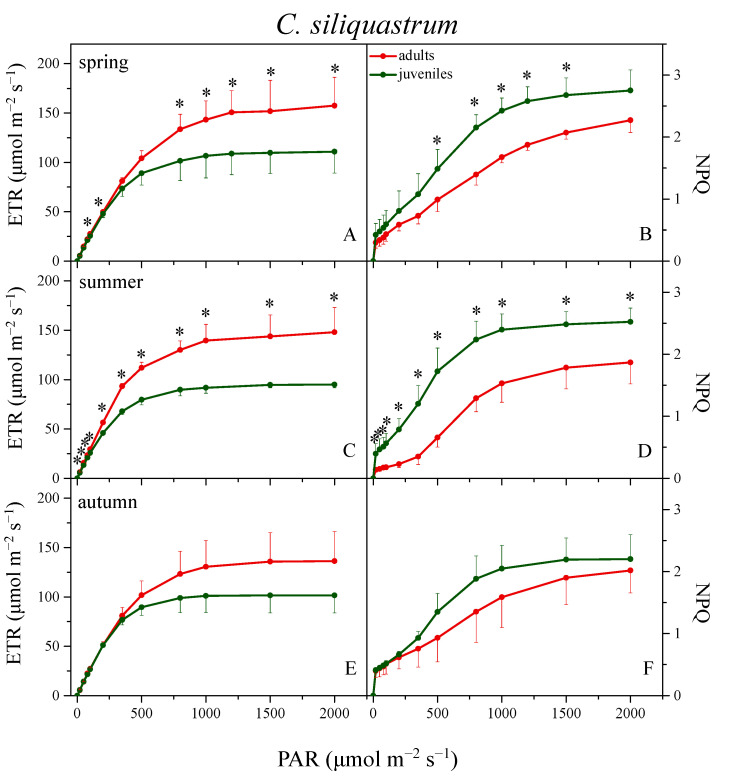
Light response curves of linear electron transport rate (ETR, left panels) and non-photochemical quenching (NPQ, right panels) from adults (red circles) and juveniles (green circles) of *C. siliquastrum* during spring (**A**,**B**), summer (**C**,**D**), and autumn (**E**,**F**). Data are means ± SD of 6 measurements (2 measurements/individual). Asterisks denote statistically significant differences (*p* < 0.05) between adults and juveniles at each light intensity within each season.

Figure 2 presents the light response curves of calculated electron transport rate (ETR, left panels) and non-photochemical quenching (NPQ, right panels) from adults and juveniles of *N. oleander*, for each distinct season. Juveniles showed significantly lower ETR values than adults in almost all PAR intensities and all seasons, except autumn, with the differences intensifying during summer and winter. In adults, the ETR showed higher values in spring, declined slightly during summer (~20% at the high PAR intensities), recovered after the autumnal rains, and reached the lowest levels (i.e., ~40% reduction at all light intensities compared to spring) during winter. The corresponding juveniles followed a similar seasonal pattern, yet the magnitude of decline in summer and winter was much higher (45 and 60%, respectively). In autumn, the ETR of juveniles increased substantially (ca. 40% relative to spring), thus reaching the adult values. In *C. siliquastrum*, no significant variation in ETR was observed among seasons for both adults and juveniles (Figure 3, left panels). Juveniles showed a significantly lower ETR in spring and summer, at almost all light intensities, while during autumn the same trend was not statistically significant.

Concerning NPQ, adults and juveniles of *N. oleander* showed the highest values in summer, intermediate in spring and autumn, and, surprisingly, the lowest values were observed during winter (Figure 2, right panels). Despite the differences in NPQ_max_ between adults and juveniles not being that large during spring and summer, juveniles displayed ca. 2–3 fold higher values than adults at the intermediate and lower PAR intensities. In autumn, the above differences were eliminated while the slight superiority of adults in winter was not statistically significant. In the case of *C. siliquastrum*, both ages showed slight variation in NPQ among seasons. Compared to adults, NPQ of juveniles was significantly higher at all light intensities in spring and especially in summer, while during autumn, as in the case of ETR, the difference was markedly reduced (Figure 3, right panels).

Maximum quantum yields of PSII activity (F_v_/F_M_) obtained during the RLC protocol provide zero ETR values, as they are extracted in the absence of light. However, the comparison of F_v_/F_M_ values between adults and juveniles on a seasonal basis can be found in a previous study of our group [5].

### 3.2. Photosynthetic Parameters after RLC Fitting

Rapid light curves (RLCs) provide information on the saturation characteristics of electron transport regarding the short- or long-term photoacclimation to light fluctuations. In the present study, we used a relatively long (30 s) period of exposure to each actinic light step instead of the most common protocol with 10 s intervals. Although the steady state conditions of photosynthesis under a specific light intensity needs 2–3 min for conventional light curves (LC), RLC protocols have been extensively studied and their usefulness in comparing photo-acclimation capacity of photosynthesis among species and/or environmental conditions has been confirmed [36,43,44].

Photosynthetic parameters obtained from RLC fitting (cardinal points) are presented in Table 1. The ETR_max_, which is related to photosynthetic capacity, in adults of *N. oleander* was highest in spring (174.2 μmol m^−2^ s^−1^), while in the corresponding juveniles in autumn (159.4 μmol m^−2^ s^−1^). Reduction in ETR_max_ in the stressful periods of summer and winter was evident for both adults (136.5 and 92.1 μmol m^−2^ s^−1^) and juveniles (99.8 and 34.5 μmol m^−2^ s^−1^). Reduction in winter was more severe than in summer for both adults and juveniles, however, values were 40–65% lower in juveniles through all seasons, except in autumn. In autumn, ETR_max_ was similar and high in both age classes (154.5 and 159.4 μmol m^−2^ s^−1^, respectively). In *C. siliquastrum*, ETR_max_ did not change significantly among seasons, yet it was always lower in juveniles (25–35%), compared to adults. The E_m_ values indicate that adults of *N. oleander* reach the ETR_max_ at high light intensities (1540–1930 μmol m^−2^ s^−1^) in all seasons, while E_m_ values in juveniles were almost half those of adults during summer and winter. This reduction was also evident in *C. siliquastrum*, yet of lower magnitude. Minimum saturation irradiance (E_k_) marks the minimum light energy, necessary for a plant to saturate photosynthesis, that is, to efficiently photo-acclimate during transition from the light-limited to the light-saturated conditions. E_k_ for adults of *N. oleander* ranged between 515 and 660 μmol m^−2^ s^−1^ PAR while for juveniles was only ~375 μmol m^−2^ s^−1^, except autumn where the Ek value was significantly higher (568 μmol m^−2^ s^−1^). Analogous, but of lower magnitude, differences were found between adults and juveniles of *C. siliquastrum*.

### 3.3. Photosynthetic Pigments

The seasonal variations in area-based total chlorophyll and carotenoid content for adults and juveniles of *N. oleander* and *C. siliquastrum* are given in Table 2. In *N. oleander*, the chlorophyll concentration was almost constant among seasons in adults, while in the corresponding juveniles it was increased in autumn and remained stable thereafter. No notable difference was observed between adults and juveniles in any season. In *C. siliquastrum*, no significant seasonal variation in total Chls was observed for both age classes, yet juveniles always showed ca. 40% lower chlorophyll concentration compared to adults.

Total carotenoids increased gradually from summer to winter in adults and juveniles of *N. oleander*, with no difference between them through seasons. In *C. siliquastrum*, total carotenoids were almost stable seasonally in adults, while in juveniles they increased only during the summer. In addition, juvenile values were always ~40% lower than the corresponding adult.

**Figure 4:**
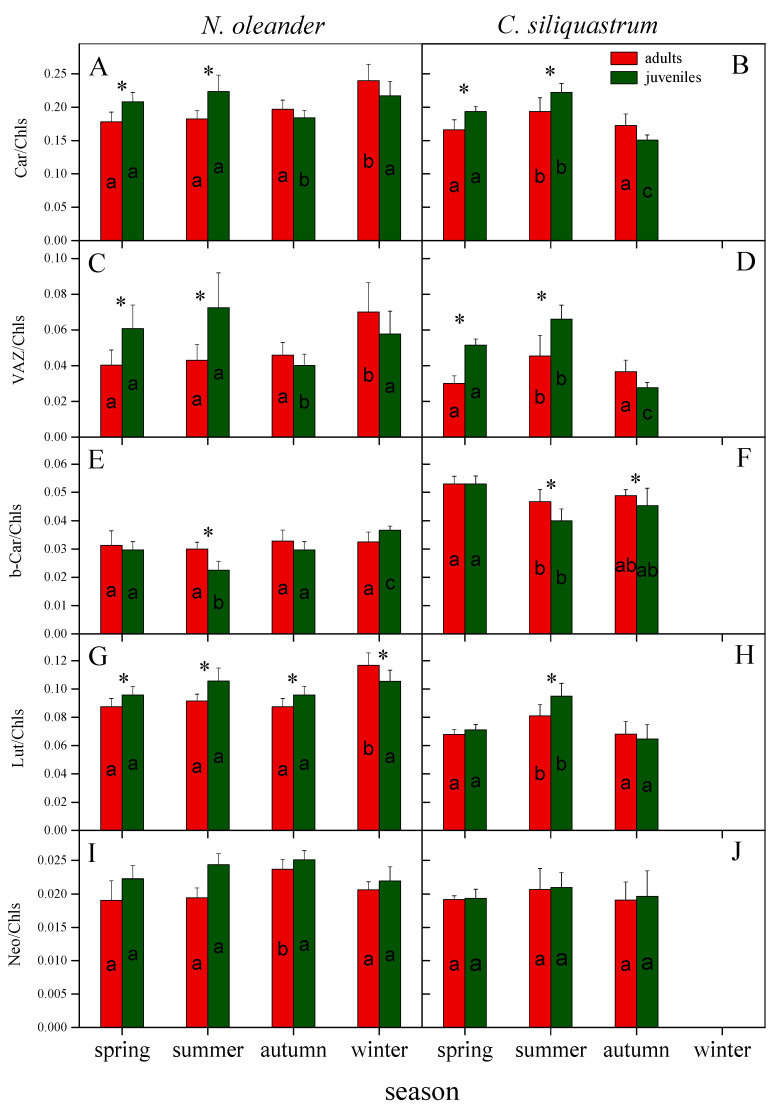
Seasonal variation in total carotenoids (**A**,**B**), xanthophyll cycle components (**C**,**D**), b-carotene (**E**,**F**), lutein (**G**,**H**), and neoxanthin (**I**,**J**) on a chlorophyll basis (μg μg^−1^ Chl) for adults (red columns) and juveniles (green columns) of *N. oleander* (left panels) and *C. siliquastrum* (right panels). Data are means ± SD of 6 independent extractions. Within each species, asterisks denote statistically significant differences (*p* < 0.05) between adults and juveniles for the indicated parameter in the same season, while different letters indicate statistically significant differences (*p* < 0.05) of adults or juveniles between seasons. VAZ: violaxanthin + antheraxanthin + zeaxanthin.

Expressing total carotenoids on a chlorophyll basis (Car/Chls), both plant species followed a similar pattern, that is juveniles displayed 15–20% higher values than adults during spring and summer. Regarding the seasonal fluctuation of the corresponding Car/Chls ratio for each age class, an increase was observed in adults of *N. oleander* during winter and a decrease in juveniles during autumn. In *C. siliquastrum*, Car/Chls ratio increased in summer for both adults and juveniles and decreased in autumn in juveniles (Figure 4A,B).

According to our chromatographical data, the higher Car/Chls ratio in juveniles of both species is mainly shaped by the increased pool size (~60% on average) of the xanthophyll cycle components (VAZ/Chls) in spring and summer, implying an enhanced photoprotective demand already from the favorable season of the year. The seasonal fluctuation of VAZ/Chls ratio was analogous to that of total carotenoids, yet the magnitude of differences between adults and juveniles was higher (Figure 4C,D).

**Figure 5:**
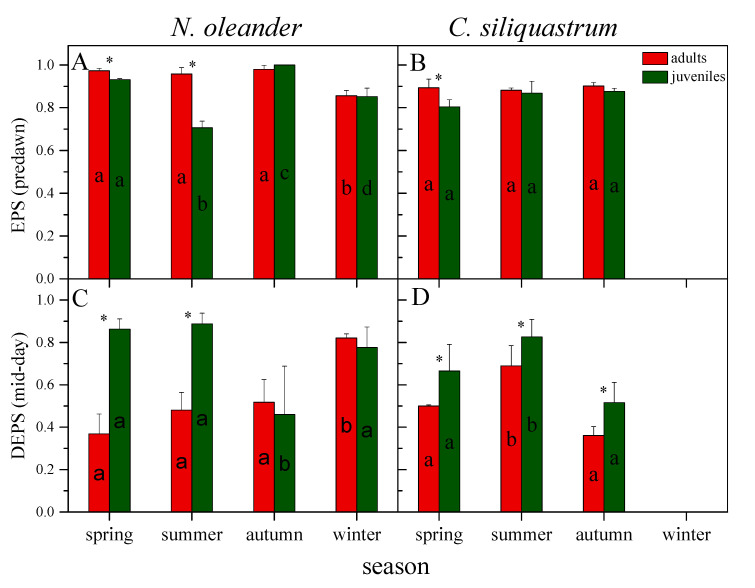
Seasonal changes in predawn epoxidation state (EPS) and midday de-epoxidation state (DEPS) for adults (red columns) and juveniles (green columns) of *N. oleander* (**A**,**C**) and *C. siliquastrum* (**B**,**D**). Data are means ± SD of 3 independent extractions. Within each species, asterisks denote statistically significant differences (*p* < 0.05) between adults and juveniles in the same season, while different letters indicate statistically significant differences (*p* < 0.05) of adults or juveniles between seasons. EPS = (V + 0.5 A)/VAZ, DEPS = (Z + 0.5 A)/VAZ.

The enhanced VAZ cycle pool size of juveniles is combined with higher DEPS (mid-day de-epoxidation state) values either in spring and summer (*N. oleander*) or in all seasons (*C. siliquastrum*), indicating a more active cycle in juveniles under high light conditions. In adults of *N. oleander*, DEPS was increased considerably in winter while in juveniles decreased in autumn. In *C. siliquastrum*, both adults and juveniles displayed the highest values in summer and the lowest in autumn. Judging from the high EPS (epoxidation state) values, the dark reversion of zeaxanthin back to violaxanthin was almost full in adults of both plant species up to autumn, while in *N. oleander* the EPS was slightly reduced in both age classes during winter. In juveniles, a slightly lower EPS than adults was observed in spring (for both plant species) and a significant reduction in summer for *N. oleander*, possibly indicating that part of zeaxanthin is retained during the night (Figure 5).

The Chl-based content of the remaining (non-xanthophyll cycle) carotenoids showed a slight seasonal variation in both plant species (Figure 4E–J). Concerning neoxanthin, no statistically significant differences were observed between adults and juveniles in any season, while b-carotene was lower in juveniles than adults during summer (in both species) and in autumn (in *C. siliquastrum*). Finally, compared to adults, lutein levels were higher in juveniles either in summer (*C. siliquastrum*) or up to autumn (*N. oleander*).

Please note that, in both species and age classes, no significant diurnal variation was recorded in the VAZ cycle pool size and the levels of the remaining carotenoids (lutein, b-carotene, neoxanthin). Thus, data in Figure 4 are mean values from dark and high light conditions.

## 4. Discussion

Adverse environmental conditions negatively affect photosynthesis and consequently create an increased demand for photoprotection. For Mediterranean plants, the threat of photoinhibition is intensified in summer, when high irradiance is accompanied by drought stress and high temperatures, and in winter, when low temperatures coincide with high light intensities. On the contrary, spring and autumn are considered favorable seasons for photosynthesis, as high irradiance is combined with relatively abundant water supplies and mild temperatures [45]. The impact of photoinhibition on plants depends on the intensity and duration of the stress imposed in each season, as well as on the growth form of the species [3,46,47]. 

Photoinhibitory conditions cause perturbations in the supply and demand for ATP and NADPH in plant metabolism and thus the regulation of their production through photosynthetic electron transport is an important photoprotective mechanism [48]. In the present study, the data derived from rapid light-response curves of photosynthesis revealed interesting differences between adults and juveniles of both species regarding ETR and NPQ, which should not be attributed to varying light exposure since all selected plants as well as all measured leaves from each species were equally exposed to full solar radiation. RLCs provide information on photosynthesis acclimation under light-limited or light-saturated conditions and consequently for the photosynthetic capacity or the downregulation efficiency of the photosynthetic machinery [36,49]. ETR at almost all light intensities, as well as ETR_max_, was significantly higher in adults compared to juveniles in both plant species, especially in *N. oleander*. The difference was obvious in all seasons, except in autumn for *N. oleander*, while the winter deciduous *C. siliquastrum* escapes the cold season through foliage shedding. This pattern confirms the recently reported photosynthetic superiority of adults [5]. To reach ETR_max_, adults of *N. oleander* needed to face the highest irradiance levels (E_m_) in the range of 1800–1900 μmol m^−2^ s^−1^, and the correspondingly high (1550 μmol m^−2^ s^−1^) in winter. These light intensities occur only for one or two hours maximum in the field, even during the long bright days of Mediterranean spring and summer. However, on a daily basis, the period with irradiance above the saturating one (Ek), when photoprotection mechanisms are necessary, may be more critical for species survival than the short time periods with the highest irradiances. These light conditions are adverse for most of Mediterranean plant species and is a matter of tolerance for their photosynthetic machinery to overcome a short or long photoinhibitory period [27,50]. Juveniles of *N. oleander* showed significantly lower E_k_ in spring, summer, and winter compared to adults. Therefore, photosynthesis in juveniles was saturated at relatively low irradiance (~370 μmol m^−2^ s^−1^) and, accordingly, under higher light levels increased requirements of photoprotection should be expected. The corresponding E_k_ values for adults (520–660 μmol m^−2^ s^−1^) suggest that they can handle significantly higher amounts of irradiance than juveniles without the threat of photoinhibition. In *C. siliquastrum*, juveniles displayed similarly low E_k_ in all seasons, while the corresponding values for adults ranged between 500 and 540 μmol m^−2^ s^−1^. Thus, the comparison indicates that in both plant species the photoinhibitory conditions last significantly longer for juveniles than for adults during a day.

The above were confirmed through the corresponding NPQ measurements. In juveniles of *N. oleander*, the NPQ increase during spring and summer, i.e., during the periods with high light intensity and progressive development of water shortage, already reaches a maximum under low irradiance. Specifically in summer, judging by the sharp NPQ enhancement, the photoprotection need of juveniles was critical even at very low light intensity. Analogous, although less steep, differences in NPQ were also observed between adults and juveniles of *C. siliquastrum* in spring, and especially in summer. The above are consistent with previous observations that juveniles experience a significantly higher water stress impact in summer [5] and was also confirmed in the present study by the 40% (*N. oleander*) and 30% (*C. siliquastrum*) lower RWC values of juveniles compared to adults (not shown). As is known, water status reflects both the water availability in the soil and the plant’s ability to draw it up. Adults, due to their larger root system, have access to more ample water reserves in deeper soil layers, thus maintaining a higher RWC [51].

The NPQ (Stern–Volmer coefficient) reflects thermal de-excitation pathways primarily associated with zeaxanthin-dependent energy dissipation [6,16]. In our study, the enhanced NPQ values of juveniles in spring and summer were accompanied by a higher Car/Chls ratio mainly shaped by the ~50–70% increased pools of the VAZ cycle components (VAZ/Chls). Furthermore, the enhanced VAZ pool size was combined with a higher midday de-epoxidation (DEPS), indicating a more functional cycle in juveniles than adults under high light conditions. These results are consistent with previous studies showing that VAZ and AZ/VAZ are lower in older than younger plants [52] and that the increased pool and functionality of xanthophyll cycle is a common response of Mediterranean species during summer draught [53,54]. Concomitantly, juveniles of *N. oleander* showed a significant reduction of EPS (predawn) in summer, indicating that part of zeaxanthin is retained in the dark. Overnight retention of A + Z under water and/or heat stress, also reported in other Mediterranean evergreen species, may contribute to the stabilization of thylakoid membranes and/or ensure the rapid activation of the VAZ cycle early in the following morning [52,55,56,57].

Apart from the higher investment in xanthophyll cycle components and the enhanced VAZ de-epoxidation state, already appearing from spring, juveniles of both species showed higher lutein (L/Chls) and lower b-carotene (b-Car/Chls) levels. Lutein is considered a key player in the quenching of ^3^Chl [18] and seems to act as an extra photoprotective mechanism when stress becomes severe, while the decrease in b-carotene could be associated with the increase in xanthophyll cycle pool size, as under water stress conditions zeaxanthin is formed in the light partly at the expense of violaxanthin and partly of b-carotene [55,58]. In addition, juveniles of *C. siliquastrum* displayed constantly lower total chlorophyll content compared to adults, which may be considered as a supplementary photo-avoidance response that alleviates overexcitation and enhances the photoprotective action of carotenoids per chlorophyll molecule [54]. It is interesting that in juveniles, which are severely stressed during summer, the photoprotective mechanisms are early activated, already from spring, to protect them from oxidative damage. In this sense, the reduced ETR in spring may act as a regulatory mechanism to balance the supply of ATP and NADPH through the light reactions with the energetic demands of Calvin cycle, thus reducing the risk of ROS formation [59].

In winter, adults and especially juveniles of *N. oleander* showed a significant decrease in electron transport rate, while the corresponding Ek values reflect needs for photoprotection analogous to those of summer. It is therefore obvious that it is harder for both age classes of *N. oleander* to overcome photoinhibition in winter and, therefore, they exhibit the lower seasonal ETR values. These results are in accordance with previous studies clearly showing that the mild Mediterranean winter is particularly stressful for evergreen sclerophylls [5,46,53,54], even more than summer stress.

On the other hand, NPQ was unexpectedly low during winter in both age classes of *N. oleander*, while xanthophyll cycle pool size (VAZ/Chls) and functionality (DEPS) either remained as high (in juveniles) or increased substantially (in adults) compared to summer. This phenomenon, which has been previously described [53,60], could be associated with the partially quenched Fm at low temperatures, as a result of the long-term PSII downregulation [61]. This, in turn, could lead to an underestimation of NPQ [60,62,63]. In addition, the main component of NPQ (qE) is ΔpH-dependent and has been correlated with the LHCII aggregation state when antenna complex is highly dissipative through the xanthophyll cycle [15]. This aggregation is mediated via PsbS protein, which acts independently of xanthophyll biosynthesis, and if absent or not functioning leads to low qE even when VAZ is relatively high. Accordingly, an active xanthophyll cycle does not always correspond to a high NPQ [64,65]. Furthermore, since our recording protocol in the field does not permit the distinguishing of qE from qT and qI, changes in NPQ may be also associated with the redistribution of excitation energy between the photosystems and/or to photoinactivation processes [66]. In this sense, NPQ differentiations may not necessarily coincide with the corresponding changes in Car/Chls and VAZ/Chls ratios or DEPS.

One would expect that all differences observed between adults and juveniles during spring would be retained in autumn, as environmental conditions were equally favorable. In our case, however, the differences in NPQ and in the xanthophyll cycle between adults and juveniles were either eliminated (*N. oleander*) or diminished (*Q. siliquastrum*) in autumn. Analogous results concerning the photosynthetic differences between adults and juveniles were also reported in a previous seasonal study [5] and were associated with the increased energy requirements of reproduction occurring in adults during spring, which leads to positive feedback on photosynthesis [59].

## 5. Conclusions

The results of the present study revealed differences in electron flow activity between adults and juveniles, the magnitude of which shows seasonal fluctuations. These differences create different photoprotective demands in juveniles compared to the corresponding adults. In general, the photoprotective differentiation between adults and juveniles follow a common seasonal pattern in the two species tested, while the magnitude of differences in each distinct season is possibly species-specific and/or dependent on the plant growth form. Specifically, juveniles of both species showed a lower electron transport rate during spring, leading to an increased investment and functionality of the xanthophyll cycle, which was translated into higher NPQ. These differences were enlarged in summer, possibly due to the more intense water stress endured by juveniles. During autumn, no difference in ETR between adults and juveniles of *N. oleander* was found and, accordingly, no differences in NPQ and the xanthophyll cycle were observed. On the other hand, juveniles of *C. siliquastrum* showed a slightly lower linear electron transport rate, followed by a higher conversion state of the xanthophyll cycle. Finally, juveniles of *N. oleander* exhibited lower ETR than adults during winter, while *C. siliquastrum* escapes the unfavorable season through defoliation. Despite the difference in ETR, cold stress seems to create similar photoprotective needs between adults and juveniles in *N. oleander*.

## Figures and Tables

**Table 1:** Photosynthetic parameters obtained after the RLC fitting, according to Platt et al., 1980 [39]. ETR_max_: maximum relative electron transport rate; E_k_: minimum saturation irradiance; E_m_, irradiance at maximum photosynthesis.

		**ETR_max_**		**E_k_**		**E_m_**	
		**Adults**	**Juveniles**	**Adults**	**Juveniles**	**Adults**	**Juveniles**
*N. oleander*	Spring	174.2 ± 12.8 a	99.8 ± 7.2 a*	660 ± 45 a	373 ± 22 a*	1930 ± 120 a	1500 ± 85 a*
	Summer	136.5 ± 13.9 b	45.6 ± 6.1 b*	515 ± 33 b	375 ± 39 a*	1820 ± 110 a	950 ± 60 b*
	Autumn	154.5 ± 14.5 ab	159.4 ± 16.7 c	582 ± 31 b	568 ± 35 b	1820 ± 130 a	1940 ± 75 c
	Winter	92.1 ± 6.4 c	34.5 ± 4.4 b*	646 ± 39 a	377 ± 32 a*	1540 ± 90 b	950 ± 50 b*
*C. siliquastrum*	Spring	151.4 ± 16.7 a	114.1 ± 11.2 a*	539 ± 24 a	427 ± 26 a*	1400 ± 120 ab	1200 ± 80 a
	Summer	148.8 ± 17.3 a	95.4 ± 6.4 a*	528 ± 27 a	365 ± 35 b*	1500 ± 95 a	1400 ± 75 b
	Autumn	140.3 ± 19.2 a	102 ± 13.7 a*	501 ± 29 a	393 ± 22 b*	1350 ± 70 b	1000 ± 55 c*

Data are means ± SD of 6 independent measurements. Asterisks indicate statistically significant differences (*p* < 0.05) between adults and juveniles of each species in the same season, while different letters denote statistically significant differences (*p* < 0.05) of adults or juveniles between seasons.

**Table 2:** Seasonal changes in total chlorophyll (Chls) and carotenoid (Car) content on leaf area basis in adults and juveniles of *N. oleander* and *C. siliquastrum*.

		**Chls (μg/cm^2^)**		**Car (μg/cm^2^)**	
		**Adults**	**Juveniles**	**Adults**	**Juveniles**
*N. oleander*	Spring	45.5 ± 7.1 a	40.3 ± 6.7 a	8.8 ± 1.8 a	8.4 ± 1.3 a
	Summer	46.6 ± 12.4 a	42.2 ± 5.9 a	8.4 ± 1.9 a	8.7 ± 1.5 a
	Autumn	57.7 ± 5.3 a	61.5 ± 9.1 b	11.3 ± 0.9 b	11.3 ± 1.8 b
	Winter	50.8 ± 8.4 a	58.1 ± 9.1 b	12.1 ± 1.7 b	13.2 ± 1.7 b
*C. siliquastrum*	Spring	39.3 ± 5.2 a	21.0 ± 0.8 a*	6.5 ± 0.7 a	3.9 ± 0.1 a*
	Summer	37.9 ± 5.1 a	24.9 ± 3.7 a*	7.3 ± 1.0 a	5.3 ± 1.2 b*
	Autumn	40.9 ± 5.8 a	24.7 ± 3.5 a*	7.0 ± 0.6 a	3.9 ± 0.5 a*

Data are means ± SD of 6 independent extractions. Asterisks denote statistically significant differences (*p* < 0.05) between adults and juveniles of each species in the same season. Different letters indicate statistically significant differences (*p* < 0.05) of adults or juveniles from each species between seasons.

## Data Availability

The data presented in this study are available in the figures and tables of the manuscript.
